# Assessment of Biomechanical Advantages in Combined Anterior–Posterior Cervical Spine Surgery by Radiological Outcomes: Pedicle Screws over Lateral Mass Screws

**DOI:** 10.3390/jcm12093201

**Published:** 2023-04-29

**Authors:** Sang-Ho Kim, Ji-hyeon Kim, Ji-Won Kwon, Hak-Sun Kim, Seong-Hwan Moon, Kyung-Soo Suk, Byung-Ho Lee

**Affiliations:** 1 Orthopedic Department, College of Medicine, Yonsei University, Seoul 03722, Republic of Korea; sh082kim@gmail.com (S.-H.K.);; 2 Department of Orthopedic Surgery, Dangjin 9988 Hospital, Dangjin-si 31784, Republic of Korea

**Keywords:** combined anterior–posterior approach, cervical pedicle screw, lateral mass screw, cervical sagittal balance, subsidence, vertebral body remodeling

## Abstract

Background: The combined anterior–posterior approach has shown good clinical outcomes for multilevel cervical diseases. This work describes the biomechanical advantage of cervical-pedicle-screw fixation over lateral-mass-screw fixation in combined anterior–posterior cases. Method: Seventy-six patients who received combined cervical surgery from June 2013 to December 2020 were included. The patients were divided into two groups: the lateral-mass-screw group (LMS) and the pedicle-screw group (PPS). Radiological outcomes were assessed with lateral cervical spine X-rays for evaluating sagittal alignment, subsidence, and bone remodeling. Results: At 1 year postoperatively, the numbers of patients whose C2–C7 cervical lordosis was less than 20 degrees decreased by more in the PPS group (*p*-value = 0.001). The amount of vertical-length change from immediately to 1 year postsurgery was less in the PPS group than in the LMS group (*p*-value = 0.030). The mean vertebral-body-width change was larger in the PPS group than in the LMS group during 3 months to 1 year postsurgery (*p*-value = 0.000). Conclusions: In combined anterior–posterior cervical surgery cases, maintenance of cervical lordosis and protection of the vertebral body from subsidence were better with the pedicle-screw fixation. More bone remodeling occurred when using the pedicle-screw fixation method.

## 1. Introduction

In considering the surgical approach for multilevel cervical disease, the combined anterior–posterior approach can be an ideal option. The combined approach has shown minor complications and better clinical outcomes for multilevel cervical spondylotic myeloradiculopathy and degenerative kyphosis cases [1,2,3]. Techniques and protocols concerning multiple-stage operations for the combined approach have been developed, and it has been confirmed that certain techniques, such as posterior foraminotomy, can reduce complications such as C5 palsy or prevertebral swelling [4,5]. In the combined approach, deciding which way to proceed with posterior fixation is critical. Traditionally, posterior fixation of the subaxial cervical spine has been achieved using lateral mass screws, which have a history of successful clinical use and are commonly employed by surgeons, in the combined approach as well [6].

Recently, however, cervical pedicle screws have been proposed as an alternative in certain cases, such as poor bone quality or large defects in the posterior vertebral elements [7,8,9,10]. Previously, usage of cervical pedicle screws had been limited due to the difficulty of the insertion technique and major complications resulting from misplacements [7]. Furthermore, high misplacement rates have been found in cadaveric studies. However, with the development of insertion techniques, especially by Abumi [11], complications caused by screw misplacement have been shown to be limited in clinical studies [12]. Additionally, biomechanical studies have found the superior strength of pedicle screws, which are about four times stronger than lateral mass screws in diverse positions [13] and have advantages in load-sharing properties [14,15,16]. Furthermore, the development of navigation technology and freehand techniques with preoperative measuring by computed tomography has made it possible to minimize that risk [17,18,19].

In this study, we assessed the biomechanical advantages of cervical-pedicle-screw fixation over lateral-mass-screw fixation in combined anterior–posterior decompression and fusion cases by analyzing cervical sagittal balance, subsidence of allospacers, and bone remodeling of vertebral bodies.

## 2. Materials and Methods

This study was approved by the Institutional Review Board of the authors’ hospital (4-2020-1140). A retrospective review was performed to identify all combined anterior–posterior cervical decompression and fusion surgeries performed at our institution.

### 2.1. Patient Selection

All patients who received anterior–posterior combined cervical-decompression and -fusion surgery from June 2013 to December 2020 were included in this study, and 79 patients met the inclusion criteria. All patients had myelopathy, radiculopathy, or both, and conservative management such as oral medication, physical therapy, or nerve blocking was carried out prior to surgery for all patients. Exclusion criteria were as follows: (1) past histories, such as previous cervical trauma, infection, or malignancy, and cervical operation history; (2) loss of bony fusion; (3) less than 1 year of postoperative follow-up; and (4) inadequate radiological images. We set the formation of “bridging trabecular bone” as the criterion for fusion, leading to the exclusion of three patients. With preoperative operability check-ups, dual-energy X-ray absorptiometry (DXA) tests were carried out on women of over 65 years and men of over 70 years of age who were indicated for evaluation of bone mineral density.

The final number of patients in this study was 76, and operations were conducted in 305 segments. There were 40 patients who underwent surgery with the lateral-mass-screw fixation method (LMS group), and the total number of segments was 153. In total, 36 patients took operation by the posterior pedicle-screw-fixation method (PPS group), in 152 segments. All surgeries were performed below C2 level, and there was no case involved with fusion to the occiput level.

### 2.2. Surgical Indication and Technique

In this study, we selected patients who had undergone cervical decompression and fusion by the combined anterior–posterior approach as study objects. Our institution considered the combined approach for patients with certain indications based on previous studies [5,20,21,22,23]. Those indications contained cervical myelopathy and radiculopathy with multilevel spondylosis, severe ossification of the posterior longitudinal ligament (OPLL), congenital stenosis, and multilevel fixed kyphotic deformity. We also considered the combined approach for patients with predisposing risk factors for pseudarthrosis. These risk factors included tobacco use, osteoporosis, diabetes mellitus, dialysis, and rheumatoid arthritis. We prefer performing posterior–anterior–posterior (PAP) surgery over anterior–posterior (AP) surgery for various reasons: primarily, the advantage it offers in reducing prevertebral swelling. This preference is based on several studies and clinical observations that have demonstrated the efficacy of PAP surgery in reducing postoperative complications and improving patient outcomes [5].

Naturally, the majority of patients with multi-segmental cervical spondylosis can be effectively treated through posterior-only surgery, as our institute has previously reported. However, this approach is typically reserved for cases of cervical myelopathy with central stenosis (such as OPLL, calcium pyrophosphate dihydrate (CPPD) deposition disease, or ossification of the yellow ligament (OYL)) or cervical myeloradiculopathy with relatively preserved foraminal height. In cases where foraminal stenosis with decreased foraminal height is present, additional anterior cervical discectomy and fusion (ACDF) may be necessary to increase the disc and foraminal heights through insertion of allospacers. This approach can provide additional direct and indirect foraminal decompression through foraminotomy–uncovertebrectomy and foraminal-height increases. Furthermore, the decision-making process in surgery can potentially be influenced by the national healthcare service in the authors’ country [4].

In our study, there were 5 cases of severe OPLL with spondylosis and 1 case of 5-level herniated cervical disc with uncovertebral hypertrophy, and all the other cases were of severe cervical spondylotic myelopathy and radiculopathy on two or more vertebral levels and with rigid kyphosis. Patients with only predisposing risk factors for pseudoarthrosis and not belonging to the above diagnoses were not included in this study.

Preoperative vascular-enhanced computed tomography (CT) scans were carried out for all patients to assess the vascular anomaly and cervical bone structure and decide the fixation method. If the diameter of a pedicle was less than 3.5 mm or an anomaly of a vertebral artery was present in CT scans, posterior fixation with lateral mass screws was performed [24].

The operation was performed in two stages. First, decompression and posterior screw fixation were carried out via the posterior approach. The cervical kyphosis of the patient was measured before surgery and, if necessary, manually corrected using a traction system or a Mayfield head holder. During this step, we harvested auto-lamina bone by laminectomy. The posterior-fixation method was selected between lateral mass screws (VERTEX^®^ Reconstruction System, Medtronic Sofamor Danek, Inc.; Memphis, TN, USA) and pedicle screws (POSEIDON™ Occipital and Cervical Posterior Fixation System, Medyssey USA, Inc.; Buffalo Grove, IL, USA) based on the preoperative CT scans, as mentioned above. Both fixation methods were performed with freehand techniques [18,25,26]. In cases of severe deformities, the O-arm could be used in conjunction with our institution’s own techniques.

The period between two stages was determined by a patient’s general condition and could range from 3 days to 1 week. During this period, the patients were at absolute rest and wore cervical braces continuously to maintain cervical alignment. In the second stage, cervical discectomy and allospacer insertion proceeded via the anterior approach. The posterior longitudinal ligament and one-fourth of the uncinate process were removed in this stage [27]. The proper size of each cervical bone allospacer (CORNERSTONE ^®^ PSR, Medtronic Sofamor Danek, Inc.; Memphis, TN, USA) was selected to be between 6 and 7 mm in height and 12 and 14 mm in anterior–posterior diameter, depending on the original disc height and tension after insertion of the allospacer. The standard lordotic angle of the allospacer we used was 6 degrees. The allospacer was typically inserted during performing surgery on the C4-5-6-7 level, and, if needed, when C3-4 operations took place. Trial insertion was conducted under guidance of a C-arm during surgery to prevent overdistraction of the segment or insertion of oversized allospacers.

At last, in the prone position, rods were applied through the previous incision with great care, with the curvature of the rods being determined by the patient’s cervical lordosis. Posterolateral fusion using auto-lamina bone, which was harvested during decompression, was then performed.

### 2.3. Evaluation of Radiological Outcomes

**Figure 1:**
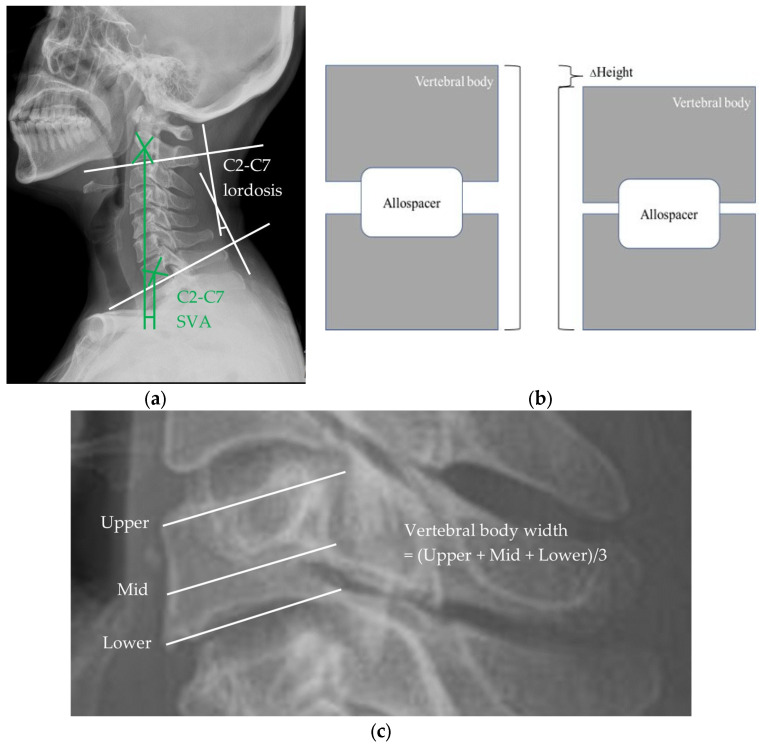
Measurements of parameters on lateral cervical radiograph: (**a**) C2–7 cervical lordosis (CL) and C2–7 sagittal vertical axis (SVA); (**b**) vertical height change; (**c**) and vertebral body width schemes following the same formatting.

This study used lateral X-rays of the cervical bone, taken in a standing position, with strict rules followed by radiologists. Technical factors included an 8 × 10 inch image receptor, a 70–80 kVp range, a mAs of 28, and a minimum SID of 60–72 inches. Positioning involved centering the midcoronal plane to the midline of the cassette, adjusting the shoulders to lie in the same horizontal plane, and asking the patient to elevate the chin slightly. The central ray (CR) should have been perpendicular to the cassette and would be directed horizontally to C-4 (level of upper margin of thyroid cartilage). Evaluation criteria included demonstrating C-1 through C-7 cervical vertebral bodies, C7-T1 junctions, intervertebral disc spaces, articular pillars, spinous processes, and apophyseal joints, with no evidence of rotation (by superimposition of both rami of the mandible, both side apophyseal joints, and the posterior borders of the vertebral bodies and optimal exposure of soft tissues and bony vertebrae. Lateral cervical spine X-rays were taken for all patients at the preoperative, immediately postoperative, 3-month follow-up and 1-year follow-up periods [28]. Screw-related complications in the subaxial cervical spine, such as pseudoarthrosis or screw pull-out, were statistically similar between the two methods, according to Yoshihara et al [29]. Therefore, the difference in radiological change could not be attributed to screw-related complications. We investigated two cervical sagittal parameters, allospacer subsidence, and vertebral body remodeling from the X-rays. Figure 1 shows the measurement method for each parameter.

The C2–C7 cervical lordosis (CL) and C2–C7 sagittal vertical axis (SVA) were used for cervical sagittal balance [30]. Improvement in cervical sagittal balance has been proven to be important in prevention of adjacent segment disease requiring revision surgery and has been shown to have better clinical outcomes [31,32,33]. The C2–7 CL was measured with Cobb’s method: the angle formed by the perpendicular lines parallel to the inferior endplates of C2 and C7 [34]. We defined the C2–7 SVA as the distance between the C2 plumbline and the posterosuperior corner of C7, as in previous studies [34].

Subsidence was defined as a decrease in the vertical length from the superior endplate of the most superior vertebra to the inferior endplate of the most inferior vertebra [35]. To calculate the subsidence of the allospacer, control values of vertical length were measured using lateral X-rays taken immediately after surgery on both groups. We considered that there was clinical subsidence when the decrease in vertical length was more than 3 mm.

We defined a new concept, “Vertebral body width”, for radiological and quantitative evaluation of vertebral body remodeling. As shown in Figure 1c the mean of the anteroposterior lengths in the upper, middle, and lower levels was measured for all of the operated vertebrae, and the average of all these measured results was finally designated as “Vertebral body width”. As a result, it meant the average anteroposterior length of the operated vertebrae. We initially considered using CT-imaging modality for measuring vertebral body width; however, it was not suitable for accurately assessing osteophyte location, and the resorption pattern was asymmetric due to the direction of insertion of the allospacer (Supplementary Figure S1). Therefore, we ultimately decided to use X-ray-imaging modality for measuring. In this study, we determined that remodeling occurred when the width changed. If the width decreased, it could be judged that resorption-dominant bone remodeling had occurred. Therefore, after obtaining of the width at each time point, the difference in the change amount was evaluated between the two posterior-fixation methods. Since there was a lack of previous studies about radiological evaluation of bone remodeling, we used the concept of change of vertebral body width.

To assess intraclass correlation, another musculoskeletal radiologist and another spine surgeon who were blinded and not related to this study also analyzed the parameters in the lateral cervical X-rays, and the intraclass correlation coefficient was calculated. Since the intraclass correlation coefficient was calculated to be higher than 0.90, the correlation was judged to be excellent in this study.

### 2.4. Statistical Analysis

An independent t-test and a chi-square test were used to evaluate the differences of the basic radiological parameters and demographic factors between the two groups. Fisher’s exact test was also used to evaluate the difference in the ratio of clinical subsidence between the two groups. All statistical analyses were performed using the SPSS 25.0 statistics package (SPSS, International Business Machines Corp., New York, NY, USA). *p*-values of less than 0.05 were considered statistically significant.

## 3. Results

### 3.1. Patient Dynamics

**Figure 2:**
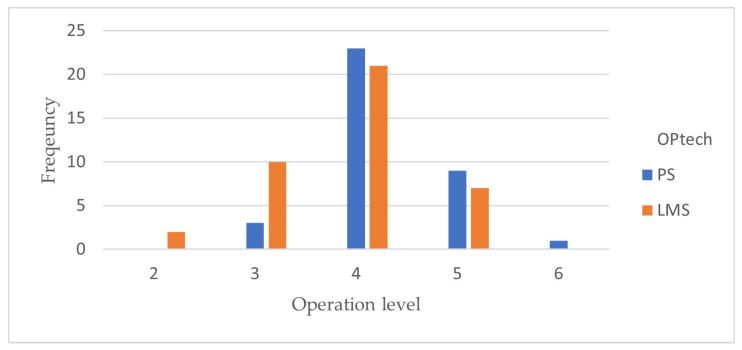
Operation-level frequency between PPS and LMS groups. PPS, the pedicle-screw; LMS, the lateral-mass-screw.

The demographic characteristics of both the LMS and PPS groups are summarized in Table 1. The ratio of male patients was 52.5% in the LMS group and 50% in the PPS group, which showed no statistical difference (*p*-value = 0.828). There was also no statistical difference in the mean age of both groups, which was 61.1 (*p*-value = 0.997). In both groups, the four-level fixation was the most common, and there was no statistically significant difference (*p*-value = 0.119) in Table 1 and Figure 2. There was only one case with complications: vocal cord palsy and aspiration pneumonia occurring after surgery.

### 3.2. Cervical Spine Sagittal Balance

Radiological parameters for the cervical sagittal balance of the two groups are summarized in Table 2. The two groups did not differ significantly in the C2–C7 SVA during the preoperative (*p* = 0.408), 3-month postoperative (*p* = 0.616), or 1-year postoperative period (*p* = 0.755).

The mean C2–C7 cervical lordosis angle was higher among the PPS group compared to the LMS group during the preoperative (*p* = 0.006), 3-month postoperative (*p* = 0.020) and 1-year postoperative periods (*p* = 0.012). In both groups, the mean C2–C7 cervical lordosis angle at 3 months postoperatively increased and then decreased slightly in the 1-year postoperative period.

The numbers of patients whose C2–C7 cervical lordosis was less than 20 degrees before the operation were 30 and 37 in the PPS group and the LMS group, respectively, and there was no significant difference (*p* = 0.294). However, at 3 months postoperatively, the numbers decreased to nine patients and twenty-two patients in each group, respectively, which showed a significant difference and greater improvement in the PPS group (*p* = 0.008). In addition, in the 1-year postoperative period, the PPS group remained at nine patients, but the LMS group slightly increased, to twenty-five patients, and there were statistically significant differences (*p* = 0.001).

### 3.3. Subsidence

Information about changes in vertical length of vertebral bodies is shown in Table 3. In the case of the PPS group, the average control value is 86.1 mm, and it is 77.5 mm in the LMS group.

The amount of vertical-length change was 1.1 ± 1.5 mm in the PPS group and 3.1 ± 4.6 mm in the LMS group at 3 months after surgery, showing a statistical difference (*p* = 0.010). From immediately to 1 year postsurgery, the change was 1.3 ± 1.4 mm in the PPS group and 3.2 ± 5.1 mm in the LMS group (*p* = 0.030). During the above two periods, the decrease in vertical length was larger in the LMS group than in the PPS group. However, there was no significant difference between the two groups from 3 months to 1 year postsurgery (0.3 ± 1.1 mm vs. 0.1 ± 2.3 mm, *p* = 0.713). The proportion of clinical subsidence was also higher among the LMS group compared to the PPS group from the immediately postoperative period to the 1-year postoperative period (47.5%; nineteen of forty patients versus 25.0%; nine of thirty-six patients, *p* = 0.042).

### 3.4. Vertebral Body Width

The average anteroposterior width of the vertebral body immediately after operation was 21.5 mm in the PPS group and 22.1 mm in the LMS group, as can be seen in Table 4.

The mean vertebral-body-width change was 1.4 ± 1.4 mm in the PPS group and 1.3 ± 2.9 mm in the LMS group at 3 months postsurgery (*p* = 0.879). The mean change was 3.0 ± 1.2 mm in the PPS group and 2.0 ± 2.8 mm in the LMS group from immediately to 1 year postsurgery (*p* = 0.054). There was no significant difference between the two groups.

However, the mean vertebral-body-width change was 1.6 ± 0.6 mm in the PPS group and 0.7 ± 0.7 mm in the LMS group at 3 months to 1 year postsurgery. There were significant differences between the two groups (*p* = 0.000) in Table 4.

## 4. Discussion

The goals of spinal surgery for cervical degenerative diseases with multisegmental neuropathy are nerve decompression, restoration of normal anatomical structure, stability of spinal structure immediately after surgery, and, consequently, restoration of vertebral body union and function. For these purposes, combined anterior–posterior surgery is preferred. Whether to select lateral-mass-screw insertion or pedicle-screw insertion for the posterior-fixation method depends on patient-specific anatomical considerations and the surgeon’s preference [5,36].

**Figure 3:**
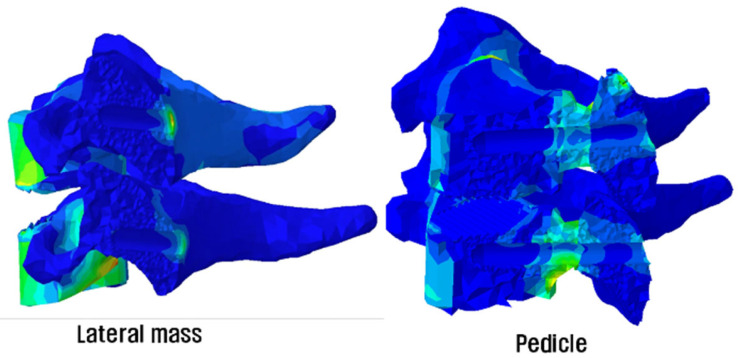
Load-sharing distribution diagram from FEM analysis (adapted from [14]).

In a previous study at our institution, Lee et al. used finite-element-model (FEM) analyses to investigate the load-sharing ratio between allograft spacers and different posterior-fixation-method combinations, which are known to be closely associated with the fusion rate. According to their study, as shown in Figure 3, the axial load was distributed throughout the vertebral body, the allospacer, and the posterior construct in the lateral-screw fixation group, whereas in the pedicle-screw fixation group, the load was concentrated on the posterior construct. This finding is closely related to our study results [14].

In this study, we compared the sagittal alignments of two posterior-fixation methods. Sagittal alignment of the cervical spine contributes to pathogenesis of cervical myelopathy, as malalignment can lead to greater cord tension, an increase in intramedullary pressure, and cord flattening, resulting in a compromised cord nature [37]. There were no case reports or studies about differences in sagittal alignment between the two methods or posterior compartment instability in the combined approach.

There was no statistical difference in the C2–C7 SVAs between the two groups. There was an increase in SVAs on both groups. One possible reason for the increase in SVAs observed in our case series could be attributed to a predominant caudal fixation approach. As the majority of distal fusion levels were at C7, it is plausible that the development of distal junctional kyphosis, particularly at the C7–T1 level during the follow-up period, may have contributed to the observed increase in SVAs postoperatively [38]. We also compared cervical lordosis between the two groups for each time period. Cervical lordosis was greater in the PPS group than in the LMS group at the preoperative period, and also larger at the 3-month and 1-year postoperative periods. It seems that there was no clinically significant difference in cervical sagittal parameter. However, we should focus on the ratio of abnormal cervical lordosis range (<20 degrees). The ratio was relatively higher in the LMS group after 3 months, and became even greater at 1 year, in contrast to the PPS group. This suggests that the ability to maintain cervical sagittal balance differed between the two groups, so we should consider pedicle screws for the posterior-fixation method when cervical sagittal disruption has already progressed or is likely to progress in the operation case.

**Figure 4:**
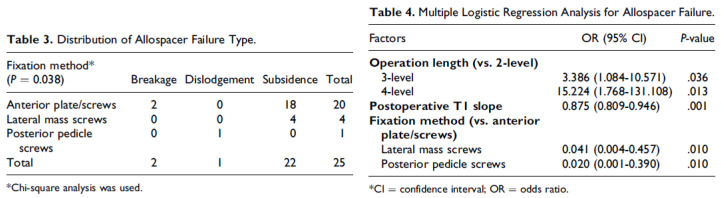
Allospacer failure distribution and statistical analysis in three fixation methods (adapted from [40]).

In the vertebral-body-fusion process after operation, subsidence has a large influence by changing spinal geometry. It is defined as the sinking of a body with a higher elasticity modulus into a body with a lower elasticity modulus. This phenomenon induces narrowing of interbody space and, consequently, general kyphosis of the spine. This may cause destabilization of the screw–plate and/or screw–bone interfaces (e.g., pulling-out, altered angulation, or breakage of the screws), so the operator should consider the fixation method that can reduce the possibility of subsidence [39]. Suk et al. reported allospacer failure distribution and statistical analysis in anterior plate/screw fixations, lateral-mass-screw fixations, and pedicle-screw fixations, as shown in Figure 4. In this previous study’s results, pedicle-screw fixations showed lower odds ratios than lateral-mass-screw fixations, including in clinical subsidence [40].

Kwon et al. reported that the stress of the pedicle-screw–rod construct was evenly distributed throughout the three columns of the cervical vertebra through FEM analysis [15]. They found that the peak von Mises stress (PVMS) on the allospacer in the lateral-mass-screw construct was higher than the PVMS in the pedicle-screw construct. Additionally, Dunlap et al. also reported that, in a cadaveric experiment, cervical pedicle-screw–rod constructs demonstrated greater reductions in axial load transfer through the intervertebral disc than lateral mass screw–rod constructs [16].

In this study, we found that the decrease in vertebral vertical length was higher in the lateral-mass-screw fixation cases for 3 months after operation. The proportion of cases in which the vertical length decrease was more than 3 mm was also larger in lateral-mass-screw fixations. After 3 months, the rate was similar, as the union of the anterior compartment was completed at that time. Therefore, our study results corroborate those of previous studies.

The reason explaining this difference is the load-sharing ratio. As previously mentioned, according to Lee et al., a lateral-mass-screw fixation transfers the load to the vertebral body, while a pedicle-screw fixation has a solid screw–rod construct. Since the pedicle screw supports up to the front of the body, the load is not applied to the vertebral body and is transmitted along the screw–rod construct [14]. Based on our studies, surgeons should consider pedicle-screw fixation methods in cases of osteoporotic vertebral bodies or instability in the anterior compartment.

**Figure 5:**
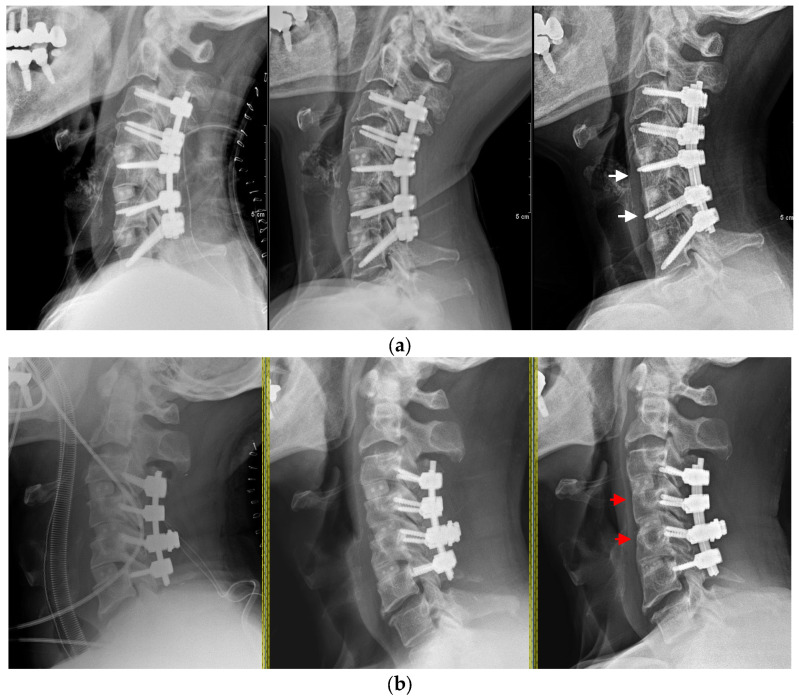
Vertebral remodeling patterns. (**a**) Immediately post-op/3-month follow-up/1-year follow-up lateral X-rays of PPS-group cases. White arrows (→) show vertebral body remodeling. (**b**) Immediately post-op/3-month follow-up/1-year follow-up lateral X-rays of LMS-group cases. Red arrows (→) show vertebral body remodeling.

As shown in Figure 5, there was no significant difference in the vertebral body width between the two groups immediately after surgery and at 3 months postsurgery. However, in the follow-up between 3 months and 1 year postsurgery, not only the absolute difference in width but also the rate of change in width showed a statistical difference.

As mentioned earlier, this difference can be explained to be due to the degree of load according to the biomechanical difference of the two posterior fixing screws [14]. The pedicle screw was inserted up to 2 mm from the anterior tip of the vertebral body, and thus, the pedicle screw and rod supported more significant parts of the physiological loading than the vertebral body and the allograft spacers. As a result, the stress-shielding effect occurred in the anterior part of the cervical spine, especially in the vertebral body and the allospacers, which led to bone remodeling in the form of bone resorption. On the other hand, with the lateral mass screw, more of the cervical load was carried by the vertebral body and allograft constructs than the pedicle-screw–rod construct, and hence, bone remodeling was relatively less. As there was no previous clinical study of difference in the stress-shielding effect between the two fixation methods, this finding provides reasonable evidence of differences in load-sharing between the two fixation methods clinically.

This study has limitations. Firstly, the number of study cases was not large, and the maximum follow-up period was one year, which is a relatively short-term follow-up. However, considering that there are generally few combined anterior–posterior-approach cases for severe myeloradiculopathy, and that the pedicle-screw-insertion technique is not the traditional method, this limitation was inevitable. Secondly, in the analysis of the sagittal parameter, the preoperative sagittal parameters showed statistical differences between the two groups, which might have affected the results of our study. The studied patients with severe spondylosis and kyphosis underwent combined anterior–posterior surgery with lateral-mass-screw fixation, as the deformation and sclerosis of the pedicle precluded the insertion of pedicle screws. Therefore, these patients had to be assigned to the LMS group relatively more, which could explain such results. However, as explained in the discussion section, there was a difference in maintaining cervical lordosis between the two methods, and there was no study proving that the initial value affects maintenance of lordosis. Therefore, the evaluation of cervical lordosis was statistically significant.

The strength of this study was that it is the first study showing differences in the stress-shielding effect by load-sharing distribution, enough to demonstrate the different patterns of remodeling based on the biomechanical differences between lateral mass and pedicle screws. In particular, surgical techniques that do not involve simultaneous fixation of the anterior and posterior aspects are uniquely performed in Korea. Therefore, the value of this paper is high, as it enabled us to exclude the effect of anterior fixation when analyzing the biomechanical differences caused by posterior-fixation methods. Further comparative studies on long-term follow-up and complex studies including various causative factors that may affect the occurrence of graft failure or subsidence not considered in this study are needed in future studies.

## 5. Conclusions

In combined anterior–posterior cervical surgery cases, maintenance of cervical lordosis and protection of the vertebral body from subsidence were better with the pedicle-screw fixation than with the lateral-mass-screw fixation. Furthermore, more bone remodeling as a result of resorption occurs in the anterior portion of the vertebral body–allograft spacer constructs when the pedicle-screw fixation method is used, which can be explained by the stress-shielding effect. Compared to the lateral mass screws, posterior constructs with pedicle screws shared a more physiological load.

## Figures and Tables

**Table 1:** Comparison of demographic characteristics between the two groups.

	PPS Group (*n* = 36)	LMS Group (*n* = 40)	Overall	*p*-Value
Sex Male Female	18 (50%) 18 (50%)	21 (52.5%) 19 (47.5%)	39 (51.3%) 37 (48.7%)	0.828
Age	61.1 ± 11.0	61.1 ± 11.6	61.1 ± 11.3	0.997
Operation Level 2 3 4 5 6	0 (0.0%) 3 (8.3%) 23 (63.9%) 9 (25.0%) 1 (2.8%)	2 (5.0%) 10 (25.0%) 21 (52.5%) 7 (17.5%) 0 (0.0%)	2 (2.6%) 13 (17.1%) 44 (57.9%) 16 (21.1%) 1 (1.3%)	0.119

**Table 2:** Comparison of sagittal alignment between the two groups.

	PPS Group (*n* = 36)	LMS Group (*n* = 40)	Overall	*p*-Value
Pre-operative				
C2–C7 SVA (mm)	17.4 ± 10.6	19.8 ± 14.0	18.6 ± 12.5	0.408
C2–C7 Cervical Lordosis Angle	14.6 ± 11.1	7.6 ± 10.6	10.9 ± 11.3	0.006
C2–C7 Cervical Lordosis < 20 (*n*, %)	30 (83.3%)	37 (92.5%)	67 (88.2%)	0.294
3 months				
C2–C7 SVA (mm)	25.8 ± 18.7	23.9 ± 13.6	24.8 ± 16.2	0.616
C2–C7 Cervical Lordosis Angle	22.8 ± 5.7	18.3 ± 10.0	20.4 ± 8.5	0.020
C2–C7 Cervical Lordosis < 20 (*n*, %)	9 (25%)	22 (55%)	31 (40.8%)	0.008
1 year				
C2–C7 SVA (mm)	25.7 ± 17.4	24.6 ± 13.6	25.1 ± 15.4	0.755
C2–C7 Cervical Lordosis Angle	21.4 ± 6.4	16.2 ± 10.7	18.7 ± 9.2	0.012
C2–C7 Cervical Lordosis < 20 (*n*, %)	9 (25%)	25 (62.5%)	34 (44.7%)	0.001

**Table 3:** Comparison of subsidence between the two groups.

	PPS Group (*n* = 36)	LMS Group (*n* = 40)	Overall	*p*-Value
Immediately post-op				
Control for Vertical Length (mm)	86.1 ± 15.0	77.5 ± 12.7	81.6 ± 14.4	0.009
3 months				
Mean Vertical Length (mm)	85.0 ± 14.7	74.4 ± 11.7	79.4 ± 14.1	0.001
Amount of Change (Imme. to 3 mo.) (mm)	1.1 ± 1.5	3.1 ± 4.6	2.1 ± 3.6	0.010
Clinical Subsidence (>3 mm) (*n*, %)	6 (16.7%)	14 (35.0%)	20 (26.3%)	0.070
1 year				
Mean Vertical Length (mm)	84.8 ± 14.9	74.3 ± 12.0	79.2 ± 14.3	0.001
Amount of Change (3 mo. to 1 yr) (mm)	0.3 ± 1.1	0.1 ± 2.3	0.2 ± 1.8	0.713
Clinical Subsidence (>3 mm) (3 mo. to 1 yr) (*n*, %)	0 (0%)	5 (12.5%)	5 (6.6%)	0.056
				
Amount of Change (Imme. to 1 yr) (mm)	1.3 ± 1.4	3.2 ± 5.1	2.3 ± 3.9	0.030
Clinical Subsidence (>3 mm) (Imme. to 1 yr) (*n*, %)	9 (25%)	19 (47.5%)	28 (36.8%)	0.042

**Table 4:** Comparison of vertebral body width between the two groups.

	PPS Group (*n* = 36)	LMS Group (*n* = 40)	Overall	*p*-Value
Immediately post-op				
Control for Vertebral Body Width (mm)	21.5 ± 1.9	22.1 ± 3.1	21.8 ± 2.6	0.259
3 months				
Mean Vertebral Body Width (mm)	20.1 ± 1.4	20.8 ± 1.9	20.5 ± 1.7	0.056
Vertebral Body-Width Change (mm)	1.4 ± 1.4	1.3 ± 2.9	1.4 ± 2.3	0.879
1 year				
Mean Vertebral Body Width (mm)	18.5 ± 1.4	20.1 ± 2.0	19.4 ± 1.9	0.000
Vertebral-Body-Width Change Amount (3 mo. to 1 yr) (mm)	1.6 ± 0.6	0.7 ± 0.7	1.1 ± 0.8	0.000
Vertebral-Body-Width Change Amount (Imme. to 1 yr) (mm)	3.0 ± 1.2	2.0 ± 2.8	2.5 ± 2.2	0.054

## Data Availability

Not applicable.

## Supplementary Materials

The following supporting information can be downloaded at: https://www.mdpi.com/article/10.3390/jcm12093201/s1, Figure S1: The direction of insertion of the allospacer.
