# Supplementary figures and images for: Assessment of Biomechanical Advantages in Combined Anterior–Posterior Cervical Spine Surgery by Radiological Outcomes: Pedicle Screws over Lateral Mass Screws
Source: J Clin Med. 2023 Apr 29;12(9):3201. doi: 10.3390/jcm12093201 (PMC10179026; doi:10.3390/jcm12093201)

Figure S1. The direction of insertion of the allospacer.

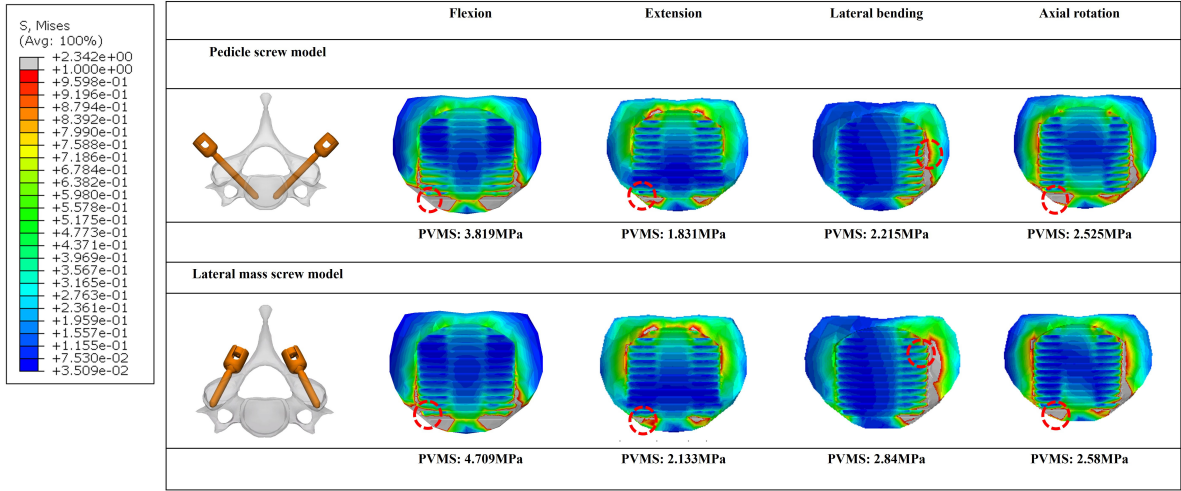

Supplement: Supplementary file 1 [file jcm-12-03201-s001.zip › jcm-2242421-supplementary.pdf]
